# Clinical Characterization of Atypical Diabetes: Insights from the GENEPEDIAB Study into the Spectrum Between Type 1 and Monogenic Diabetes

**DOI:** 10.3390/cells15050484

**Published:** 2026-03-07

**Authors:** Antoine Harvengt, Gauthier Pirlot, Leyan Denizli, Zain Syed, Sophie Welsch, Dominique Beckers, Thierry Mouraux, Nicole Seret, Marie-Christine Lebrethon, Raphael Helaers, Pascal Brouillard, Miikka Vikkula, Philippe A. Lysy

**Affiliations:** 1 EDIN Laboratory, Institute for Experimental and Clinical Research, Université catholique de Louvain (UCLouvain), 1200 Brussels, Belgium; 2 Pediatric Endocrinology Unit, Cliniques universitaires Saint Luc, 1200 Brussels, Belgium; 3 Pediatric Endocrinology and Diabetology Unit, CHU-UCL Namur Sites Sainte-Elisabeth and Mont-Godinne, 5000 Namur, Belgium; 4 Pediatric Endocrinology and Diabetology Unit, Clinique CHC MontLégia (CHC MontLégia Clinic), 4000 Liège, Belgium; 5 Pediatric Endocrinology Unit, CHU of Liège Site Notre Dame-des Bruyères, 4000 Liège, Belgium; 6 Human Molecular Genetics, de Duve Institute, Université catholique de Louvain (UCLouvain), 1200 Brussels, Belgiumpascal.brouillard@uclouvain.be (P.B.); miikka.vikkula@uclouvain.be (M.V.)

**Keywords:** type 1 diabetes, pediatrics, atypical diabetes, monogenic diabetes, whole exome sequencing, glycemic variability, biomarkers

## Abstract

**Highlights:**

**What are the main findings?**

- Our results suggest that diabetes encompasses heterogeneous clinical presentations, with features that may overlap between type 1 and monogenic forms. In this study, atypical diabetes represents the intermediate stage, thus bridging the gap between the two other forms.
- This gradient, although less statistically robust, was also observed when the atypical diabetes cohort was divided based on the number of positive DIAMODIA criteria met.

**What are the implications of the main findings?**

- These findings emphasize the clinical heterogeneity of diabetes, complicating the precise etiological diagnosis and highlighting overlap between the different forms of diabetes.
- Screening for different forms of diabetes is essential to optimizing treatment and care for patients with diabetes.

**Abstract:**

Background: Type 1 diabetes (T1D) shares clinical characteristics with other forms of diabetes, particularly monogenic diabetes such as maturity-onset diabetes of the young (MODY). Differential diagnosis is complicated by the existence of intermediate phenotypes. We aimed to delineate the phenotypic continuum between T1D and monogenic diabetes. Methods: The multicentric GENEPEDIAB study included patients aged 6 months to 18 years diagnosed with diabetes and treated for either T1D or monogenic diabetes. Analyses comprised glycemic variability, continuous glucose monitoring metrics, application of the DIAMODIA criteria, and genetic investigations. Results: A gradient was observed across T1D, atypical diabetes (Adia), and MODY cohorts for several glycemic parameters. T1D patients exhibited values furthest from treatment targets, whereas MODY patients showed better glycemic control. Stratification of the Adia cohort according to the number of positive DIAMODIA criteria further supported this trend, as demonstrated by glycemic measures and multiple correspondence analysis. Genetic analyses did not identify a uniform causative variant in the Adia cohort; however, several rare variants, including variants of uncertain significance and likely pathogenic variants in diabetes-related genes, were detected. Conclusions: These findings showed, in our specific cohort of pediatric patients, the existence of a phenotypic gradient between T1D and monogenic diabetes, with atypical diabetes occupying an intermediate position, including when stratified by DIAMODIA criteria.

## 1. Introduction

Type 1 diabetes (T1D) has long been defined as an autoimmune disease characterized by the destruction of pancreatic β-cells, which leads to insulin dependence. In pediatric populations, T1D is the most common form of diabetes, representing approximately 90% of cases. It is characterized by insulin deficiency and the presence of autoantibodies that target islet cell antigens [1,2]. However, recent advances have revealed significant clinical, immunological, and genetic heterogeneity within T1D, challenging this traditional understanding. As Battaglia et al. have highlighted, T1D is not a single disease, but rather a spectrum of distinct subtypes, or “endotypes,” driven by diverse pathogenic mechanisms. This heterogeneity is evident in variations in age of onset, disease progression, residual insulin secretion, and therapeutic responses [3].

A striking example of this complexity is the overlap in signs and symptoms between T1D and other forms of diabetes, particularly monogenic diabetes (e.g., maturity-onset diabetes of the young [MODY]) [4]. Some patients exhibit intermediate features, which complicates the differential diagnosis process. For instance, individuals with a T1D-like phenotype may lack characteristic autoantibodies, while others initially suspected of having MODY may show autoimmune involvement. These atypical cases challenge conventional classifications and highlight the need for an approach that integrates genetic, immunological, and metabolic markers [5,6].

Monogenic diabetes is rare, comprising 1–4% of pediatric cases, and is characterized by early neonatal onset (permanent neonatal diabetes mellitus and transient neonatal diabetes mellitus), hyperglycemia before age 25 (for MODY), and/or a strong familial component [2,7,8]. Unlike T1D, glycemic control is easier to achieve in monogenic forms due to relatively preserved β-cell function [9]. However, these subtypes of diabetes remain underdiagnosed due to their phenotypic similarities with T1D or type 2 diabetes (T2D) [10].

In this context, our study’s aim was to improve our understanding of pediatric patients with atypical diabetes—those whose clinical presentation falls between T1D and monogenic diabetes. We sought to identify and characterize these intermediate phenotypes through the evaluation of the DIAgnose MOnogenic DIAbetes (DIAMODIA) score. DIAMODIA score is a diagnostic tool based on clinical expertise, published evidence, and key parameters adapted from the MODY probability calculator [11]. After initially validating the score in a genetically confirmed monogenic diabetes cohort, we applied it to children and adolescents who had previously been treated for T1D in order to evaluate its clinical and genetic usefulness. We examined clinical profiles, treatment responses, glycemic control, and genetic findings to delineate the phenotypic continuum between classical T1D and MODY. By doing so, our study likely provides new insights into the precision diagnosis of pediatric diabetes. To this end, we move beyond categorical labels in order to deliver individualized, etiology-driven care.

## 2. Materials and Methods

### 2.1. Study Context

The GENEtic forms of PEdiatric DIABetes (GENEPEDIAB) study was a multicenter, retro- and prospective, interventional, and diagnostic study designed to propose a new approach for detecting atypical diabetes based on the creation of a new score. For this study, the Cliniques universitaires Saint-Luc (CuSL) collaborated with four Belgian hospitals: CHU-UCL Namur; Godinne site (Yvoir) and Saint Elisabeth site (Namur); CHU Liège, Notre Dame des Bruyères site (Liège); Cliniques CHC Mont-Légia (Liège). It is worth noting that the GENEPEDIAB study was part of the DiaType project, a multidisciplinary consortium of three Belgian universities (UCLouvain, Université libre de Bruxelles and Vrije Universiteit Brussel). The project’s primary aim was to develop personalized diabetes medicine in Belgium. The protocol was approved by the CuSL Central Ethics Committee and various local ethics committees (ethics committee study number: 2018/23JAN/023). The study was conducted in accordance with the Helsinki Declaration.

### 2.2. Patient Selection

Eligible patients for the GENEPEDIAB study were between 6 months and 18 years of age and had been diagnosed with diabetes according to the criteria of the American Diabetes Association [12]. To avoid the influence of the honeymoon period, all patients participating in the study had to have been treated for either T1D or MODY for at least 18 months. Our previous study described the complete inclusion requirements and presented the DIAMODIA criteria as a new tool for identifying patients with monogenic or atypical diabetes [11].

### 2.3. Study Procedure

The first step of the GENEPEDIAB study involved analyzing clinical and glycemic parameters previously collected from the medical records of 446 patients randomly selected from among the 1552 patients with diabetes who were being followed-up by the participating centers. All data were managed using REDCap 13.11.1 (Research Electronic Data Capture) tools provided by the Vanderbilt University (Nashville, TN, USA), and hosted at CuSL [13]. The following informations were collected: (1) patient information: gender, date of birth, country of origin; (2) patient history: birth height, weight, history of gestational diabetes, neonatal hypoglycemia history, autoimmune disease, diabetes family history; (3) anthropometric data at diabetes diagnosis: diagnosis date, age, weight, height, body mass index (BMI); (4) diabetes diagnosis testing: fasting blood glucose, 1 and 2 h OGTT (Oral Glucose Tolerace Test) blood glucose, HbA_1c_, islet autoantibodies (anti-GAD65, IA2, insulin percentage, and zinc transporter 8 [ZnT8]), human leukocyte antigen (HLA) genotype, basal C-peptide levels, residual insulin secretion, ketoacidosis defined by a venous pH < 7.35 or bicarbonate < 22 mmol/L [14]; (5) diabetes management: treatment start date, treatment types; and (6) glycemic variability represented by three measures of IDAA_1c_ and GTAA_1c_. Z-scores for height, weight, and BMI were adjusted for age and gender according to Belgian reference standards [15]. BMI score evaluation was based on the international BMI cut-offs (International Obesity Task Force) for thinness, overweight, and obesity in children and adolescents [16].

### 2.4. Glycemic Variability

Glycemic variability was assessed using two clinical scores: The insulin-dose adjusted A1c (IDAA_1c_) score and the glycemic target–adjusted HbA_1c_ (GTAA_1c_) score. Mortensen described the IDAA_1c_ score as follows: HbA_1c_ (%) + (4 × insulin dose [U/Kg body weight per 24 h]) [17]. The second clinical score was the GTAA_1c_, which our center described as follows: HbA_1c_ (%) − (3 × % of normoglycemic values [70–180 mg/dL]) [18]. For each patient, IDAA_1c_ and GTAA_1c_ scores were calculated based on three separate visits with 9 months of recorded continuous glucose monitoring (CGM) data, eventually generating an average score. A mean score of less than 9 for IDAA_1c_ and less than 4.5 for GTAA1 represented low glycemic variability and was considered a positive criterion for atypical diabetes. We also used the coefficient of variation (CV), which is an indicator of hyper- and hypoglycemia that shows the amplitude of glucose excursion [19]. The CV was calculated as follows: 100 × (standard deviation [SD]/mean glucose), and reported as % CV. The target threshold for % CV is ≤36% [20] because the frequency of hypoglycemia is increased beyond this value [21].

### 2.5. Continuous Glucose Monitoring

Raw CGM metrics from a 90-day interval were extracted from various CGM devices (i.e., Freestyle Libre 2 or 3, Abbott (Abbott Park, IL, USA); Dexcom G5, Dexcom (San Diego, CA, USA); Enlite^TM^, and Medtronic MiniMed (Minneapolis, MN, USA)).

Glycemic values < 20 mg/dL or displaying a glycemic change > 100 mg/dL in less than 5 min were considered artifacts and excluded from the analysis.

Hypoglycemia was defined as a value < 60 mg/dL, and hyperglycemia was defined as a value > 160 mg/dL for all glycemic measurements (i.e., capillary and subcutaneous).

The glycemic parameters studied were the proportion of time spent with hypoglycemia (time below the range; TBR_<60_), normoglycemia (time in range; TIR_60–160_), and hyperglycemia (time above the range; TAR_>160_), and the number of post-hypoglycemic hyperglycemia (PHH) events. A PHH event was defined as a hypoglycemic episode (i.e., interstitial glucose < 60 mg/dL) followed by a hyperglycemic episode (i.e., interstitial glucose > 160 mg/dL) within two hours. A PHH event begins when a value > 160 mg/dL is detected within 2 h after hypoglycemia. If a glycemic value < 160 mg/dL (e.g., 155 mg/dL) is identified during the PHH event, followed within 15 min by a glycemic value > 160 mg/dL, then the PHH event continues. The event ends when two consecutive glycemic values < 160 mg/dL (i.e., >15 min) are detected. PHH duration is the difference in minutes between the start and end of the event.

### 2.6. DIAMODIA Criteria

As mentioned earlier, our center developed the DIAMODIA criteria to screen patients with monogenic diabetes. These criteria were described and validated in a previous publication [11]. Criteria were classified as follows: (1) strong criteria: absence of anti-islet antibodies (anti-GAD65, IA2, insulin), lower insulin-dose adjusted A1c (IDAA_1c_) ≤ 9 (18 months after diagnosis), glycemic target-adjusted A1c (GTAA_1c_) ≤ 4.5 (18 months after diagnosis), and age at diagnosis ≤ 6 months; (2) weak criteria: first-degree relative with diabetes, C-peptide positivity (measured in the fasting state, with a threshold of 0.2 nmol/L), extra-pancreatic manifestations encompass symptoms affecting the nervous, cardiovascular, digestive, or urinary systems, absence of diabetic ketoacidosis at diagnosis, and history of neonatal hypoglycemia. A patient presenting at least one strong and one weak criterion is considered to have atypical diabetes.

### 2.7. Adia Cohort Subtypes and Sample Enrichment

To refine the analyses, the Adia cohort was divided according to the number of DIAMODIA criteria met. The DIA2 cohort included patients with two positive DIAMODIA criteria. The DIA3 cohort included patients with three positive DIAMODIA criteria. The DIA4+ cohort included patients with four or more positive DIAMODIA criteria.

In order to analyze the glycemic parameters of the different groups, we retrieved the CGM data of patients who were already included in this study. Due to the retrospective design, however, we were only able to collect CGM data for a subset of the different cohorts.

For this reason, the study also included new patients with available CGM. These patients were identified in a cohort that was retrospectively followed by the CuSL center. After analyzing their previously collected clinical and glycemic parameters from medical records, these patients were also submitted to the DIAMODIA criteria.

### 2.8. Genetic Analysis—Classic MODY Panel

All patients with atypical diabetes underwent genetic screening for monogenetic diabetes using the “classic” MODY panel. After blood sampling and DNA extraction, gene-sequencing of the *HNF4A*, *GCK*, *HNF1A*, *HNF1B*, *INS*, *KCNJ11*, and *ABCC8* genes was conducted at the Centrum Medische Genetica Antwerpen (Antwerp University Hospital—Belgium). Mutation analyses were performed using next-generation sequencing on an Illumina MiSeq sequencer (Illumina Inc., San Diego, CA, USA) after multiplex polymerase chain reaction enrichment with the MODY MASTR kit (Multiplicom N.V., Niel, Belgium). The genes were checked for a predefined coverage of at least 30× for all encoding exons, including intron/exon transition. Additional deletion/duplication studies were performed for the *GCK*, *HNF1A*, *HNF4A*, and *HNF1B* genes using MLPA (Multiplex Ligation-dependent Probe Amplification, SALSA^®^ MLPA^®^ Probemix P241-E1 MODY Mix 1, MRC Holland, Amsterdam, The Netherlands).

### 2.9. Genetic Analysis—Whole Exome Sequencing and Variant Interpretation

Whole exome sequencing (WES) was performed for patients who tested negative on the classic MODY panel. This was done in collaboration with the Institut de Duve—Groupe de Génétique Moléculaire Humaine (Prof. Mikka Vikkula’s team, UCLouvain, Brussels, Belgium). For this test, DNA was extracted from the whole blood using a Wizard genomic DNA purification kit (Promega, Madison, WI, USA) before being sequenced by Macrogen at the de Duve Institute, Belgium. Then, the exome was screened for a set of genes pertaining to the EXETER gene list (Table 1).

### 2.10. Variant Interpretation

First, we visually inspected the read alignments and removed low-quality reads. Then, we used prediction tools, including Polyphen2 (v2.2.3 build r406), MutationTaster, and combined annotation-dependent depletion (CADD), to evaluate the impact of the variants on functionality. The WES bioinformatics analysis was conducted using an in-house DNA analysis program (Highlander), with the reads aligned to the GRCh38 build of the human reference genome. The detected variants were classified using the Franklin1 platform (v90.1) [22], which applies the criteria established by the American College of Medical Genetics and Genomics (ACMG) guidelines [23]. We assessed the potential clinical impact related to the pathogenicity of the variants using public databases, including ClinVar [24], LOVD [25], and dbSNP [26], as well as data from the scientific literature available on Pubmed.gov. The variants that met our criteria were categorized into five classes: Class I, benign; Class II, likely benign; Class III, of uncertain significance; Class IV, likely pathogenic; and Class V, pathogenic. The Genome Aggregation Database (gnomAD, v4.1.0) was also consulted to determine population allele counts and number of homozygous individuals for the identified variants. We also included a consensus score indicating how many Highlander integrated networks classified the variant as damaging to evaluate the strength of the pathogenicity prediction. Variants with consensus scores above 300 or 400 (corresponding to splice- and loss-of-function mutations, respectively) were evaluated further using spliceAI (v1.3) and Exome Aggregation Consortium (ExAC, gnomAD v4.1.0) to better assess their pathogenic impact. Lastly, the variants were studied using the Rare Exome Variant Ensemble Learner (REVEL) score [27].

### 2.11. Statistical Analysis

All statistical analyses were performed using R Version 4.2.2 (R Foundation for Statistical Computing, Vienna, Austria). A *p*-value < 0.05 was considered statistically significant, with all *p*-values being two-tailed. Categorical variables were expressed as numbers and percentages, while continuous variables were expressed as means with standard deviations (SDs). The employed the Wilcoxon Rank Sum test to compare continuous variables and Pearson’s Chi-squared test and Fisher’s exact test to compare categorical variables. An exploratory multivariate analysis (multiple correspondence analysis, MCA) was conducted with the DIAMODIA score variables (categorical data) using the FactoMineR, Factoextra and Ggrepel packages. Univariate logistic regressions were applied to compare cohorts based on each DIAMODIA variable. Analysis of variance was used to assess the relationship between quantitative and qualitative variables. Then, the Bonferroni correction was employed to adjust the significance threshold (*p*-values) for multiple comparisons. The Pearson correlation coefficient was employed to measure the linear correlation between the quantitative variables and the number of positive DIAMODIA criteria.

## 3. Results

A total of 446 patients were included in the present study and divided into three cohorts: Adia (*n* = 75), Tdia (*n* = 337), and MODY (*n* = 34).

### 3.1. Comparison T1D-Adia-MODY

The first step of this study involved comparing the three cohorts (Adia, Tdia, and MODY, see Table 2).

During the neonatal period, there were significant differences in neonatal hypoglycemia and gestational diabetes between the Adia and MODY cohorts (*p* < 0.01), with a significant global *p*-value for these two parameters (*p* < 0.001). Birth height also differed between the Tdia and Adia cohorts (*p* < 0.05).

At the time of diabetes diagnosis, blood glucose levels were significantly lower in Adia patients than in Tdia patients (*p* = 0.02), but were significantly higher in the Adia cohort than in the MODY cohort (*p* < 0.001). HbA_1c_ percentage (*p* < 0.001) and body mass index (BMI, *p* < 0.01) also differed between the Adia and MODY cohorts (*p* < 0.01).

In the long term, the MODY cohort achieved better glycemic control than the Adia cohort. Glycemic parameters such as HbA_1c_ (*p* < 0.05), IDAA_1c_ score (*p* < 0.001), GTAA_1c_ (*p* < 0.001), glycemic variability (CV, *p* < 0.001), TIR_70–180_ (*p* < 0.01), and PHH duration (*p* < 0.01) were closer to target values in the MODY cohort. In contrast, patients from the Tdia cohort exhibited values distant from the treatment objectives, reflecting the more poorly controlled diabetes. Indeed, HbA_1c_, IDAA_1c_, and GTAA_1c_ values were significantly higher in the Tdia cohort than in the Adia cohort (*p* < 0.001).

In terms of diabetes treatment, the use of insulin was lower in the MODY cohort (26.5%) than in the Tdia (100%) and Adia (96%) cohorts. In fact, 19 out of 34 (55.9%) patients in the MODY cohort were not receiving any treatment, while 6 out of 34 (17.6%) were treated by oral antidiabetics alone (biguanides for 3/6 patients, sulfonylureas for 2/6 patients, and glinides for 1/6 patient). In contrast, only one patient in the Adia cohort was not receiving treatment, and two patients were treated with an oral antidiabetic agent alone (one with a sodium-glucose cotransporter 2 inhibitor and one with biguanides).

### 3.2. Division of the Adia Cohort and Subcohort Enrichment

Patients from the Adia cohort were distributed as follows: 17 patients in the DIA2 cohort, 26 patients in the DIA3 cohort, and 28 patients in the DIA4+ cohort.

CGM data were available for only 6/17 patients of the DIA2 cohort, for 6/26 patients of the DIA3 cohort, for 4/28 patients of the DIA4+ cohort, and for 2/34 patients of the MODY cohort. Partial CGM data, including mean glycemia, TIR_70–180_, TBR_70_, and TAR_180_ were retrieved for 2/17 patients of the DIA2 cohort, 1/26 patient of the DIA3 cohort, and 1/28 patient of the DIA4+ cohort. Consequently, new data from patients included in another retrospective cohort from the CUSL were added: 21 patients with CGM data were included in the Tdia cohort, five patients in the DIA2 cohort, and two patients in the DIA3 cohort. It is important to note that due to the retrospective design of the study, all CGM data comes from patients followed-up in the same clinical center (CuSL).

### 3.3. Comparisons Tdia—Adia Subcohorts—MODY

The five cohorts (Tdia, DIA2, DIA3, DIA4+, and MODY) are described and compared in Table 3.

A global statistical difference was observed at the term for neonatal hypoglycemia (*p* < 0.001) and gestational diabetes (*p* < 0.01). The only significant between-group difference was neonatal hypoglycemia between DIA4+ and MODY cohorts (*p* < 0.05).

At diagnosis, blood glucose levels (global comparison *p* < 0.001) and HbA_1c_ (overall comparison *p* < 0.001) gradually decreased from Tdia to MODY, with DIAMODIA cohorts progressively bridging the gap. Significant between-group differences were observed for DIA4+ vs. MODY (*p* < 0.05) regarding glycemia, and for DIA3 vs. DIA4+ (*p* < 0.01) as well as for DIA4+ vs. MODY (*p* < 0.05) for HbA_1c_ percentage.

Over time, MODY patients demonstrated better diabetes management compared to Tdia with, once again, the Adia subcohorts progressively making the transition. Global significant *p*-values were observed for all glycemic parameters (*p* < 0.001). For example, the closer we get to the MODY cohort, the more HbA_1c_ decreased, with all comparisons being statistically significant (*p* < 0.001, except for DIA2 vs. DIA3, *p* < 0.05), with the only nonsignificant comparison being DIA3 vs. DIA4+ cohorts (*p* = 1). This gradient was also observed for TIR_70–180_, IDAA_1c_ score, GTAA_1c_ score, CV, as well as for minimal and maximal glycemia (global comparisons *p* < 0.001). Apart from certain between-group comparisons, only the global *p*-value was statistically significant. Notably, we observed that the closer we get to the Tdia cohort, the more patients experiencing severe hypoglycemia were listed, i.e., 12/21 in the Tdia cohort vs. 0/34 the MODY cohort (global *p*-value < 0.001). A progressive decrease was also shown for PHH duration from Tdia to MODY patients, except for the DIA3 cohort (global *p* < 0.001).

Regarding diabetes treatment, we noted that all patients from the DIA2 and DIA3 cohorts were receiving insulin therapy alone. In contrast, three patients were not under insulin therapy in the DIA4+ cohort, with a patient with five criteria not under treatment, another one with five criteria treated with a sodium-glucose co-transporter 2 inhibitor (empagliflozin) alone, and the last one, who exhibited six criteria, under biguanide therapy (metformin) alone.

The five cohorts were also compared according to the DIAMODIA criteria. Since the cohorts were built based on the number of criteria met, we logically observed a gradient for the absence of anti-islet antibodies for IDAA_1c_ < 9% and for GTAA_1c_ < 4.5% (all global *p*-values < 0.001). For these three parameters, the comparison between Tdia and DIA2 cohorts was also significant (*p* < 0.001), and the comparison between DIA4+ and MODY patients was significant for the absence of anti-islet antibodies (*p* < 0.001). Indeed, the lowest values were found in the Tdia group, increased gradually in the DIA2, DIA3, and DIA4+ subgroups, and were almost always the highest in the MODY group. Moreover, only three patients were diagnosed before the age of 6 months, all from the MODY cohort. Regarding weak criteria, a gradient was observed among DIA2, DIA3, and DIA4+ for positive C-peptide secretion, family history, and absence of ketoacidosis at diagnosis (all global *p*-values < 0.001). However, we observed two outliers in the Tdia cohort. Several between-group comparisons were significant, as shown in Table 3.

### 3.4. Correlation Analysis Between Glycemic Parameters and DIAMODIA Criteria

**Figure 1:**
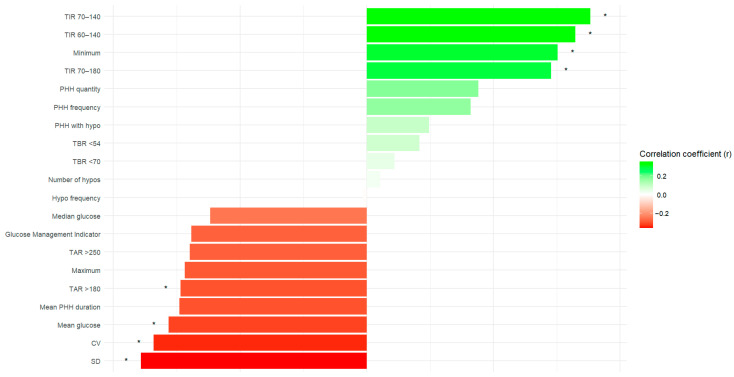
Linear correlations (Pearson’s r) between various glycemic parameters and the number of positive DIAMODIA criteria met. Significant correlations: * *p* < 0.05. DIAMODIA, DIAgnose Monogenic DIAbetes; Minimum: Minimum glycemia (mg/dL); Maximum: Maximum glycemia (mg/dL); CV, Coefficient of variation; TIR_70–180_: Time in range (70–180 mg/dL); TIR_70–140_: Time in range (70–140 mg/dL); TIR_60–140_: Time in range (60–140 mg/dL); TBR_<70_: Time below the range (<70 mg/dL); TBR_<54_: Time below the range (<54 mg/dL); TAR_>180_: Time above the range (>180 mg/dL); TAR_>250_: Time above the range (>250 mg/dL); Hypo frequency: Number of episodes of glycemia < 54 mg/dL per day; PHH: Post-hypoglycemia hyperglycemia; SD: Standard deviation.

Thereafter, we performed several linear correlations (Pearson) and demonstrated that the more DIAMODIA criteria are exhibited by a patient, the closer their glycemic parameters are to treatment objectives (Figure 1). We observed that when the number of DIAMODIA criteria met increased, CV (r = −0.34, *p* < 0.05), mean glucose levels (r = −0.31, *p* < 0.05), and TAR_180_ (r = −0.3, *p* < 0.05) decreased, while TIR_70–180_ (r = 0.29, *p* < 0.05) minimum glucose levels (r = 0.3, *p* < 0.05), TIR_60–140_ (r = 0.33, *p* < 0.05), and TIR_70–140_ (r = 0.35, *p* < 0.05) increased.

### 3.5. Multiple Correspondence Analysis

**Figure 2:**
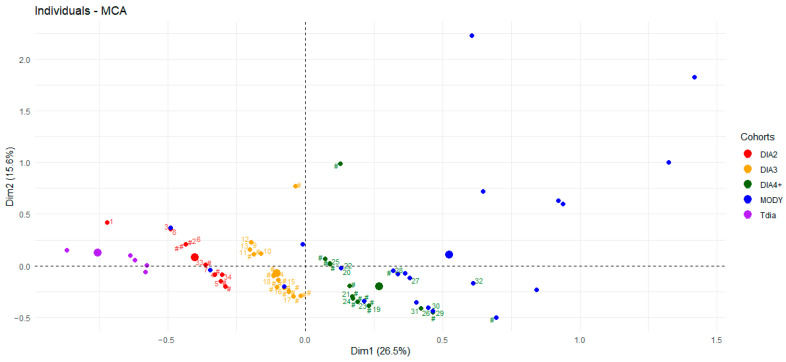
Multiple correspondence analysis of the DIAMODIA variables among the different cohorts. DIA2: Cohort of patients with two positive DIAMODIA criteria; DIA3: Cohort of patients with three positive DIAMODIA criteria; DIA4+: Cohort of patients with four or more positive DIAMODIA criteria; MODY: Maturity-onset diabetes of the young; Tdia: Type 1 diabetes. The first two MCA dimensions are displayed: Dim1 (26.5% of explained variance) on the x-axis and Dim2 (15.6%) on the y-axis. Together, these dimensions summarize the main structure of variation in the dataset. Dashed lines indicate the origin (0,0), corresponding to the average profile of all individuals.Numbers from one to thirty-four represent Adia patients for which a variant in diabetes-related genes was found and refer to Table 4. ‘#’ shows patients from Adia cohort for which any variant in a diabetes-related-genes was found.

Then, we conducted an MCA to compare the five cohorts (Tdia, DIA2, DIA3, DIA4+, and MODY) according to the DIAMODIA criteria (Figure 2). In our previous publication, we observed the formation of two distinct clusters suggesting that MODY and Tdia cohorts can be distinguished based on the DIAMODIA criteria [11]. The present analysis demonstrated that the Tdia and MODY cohorts were located at one end of the patient distribution, and that Adia patients were situated in between. The DIA2, DIA3, and DIA4+ cohorts formed a gradient within the Adia cohort, in this precise order. Therefore, our DIAMODIA criteria were not only able to distinguish MODY patients from Tdia patients, but showed that, in our study, diabetes manifests itself as a heterogenous disease whose different forms fit into a spectrum ranging from MODY to Tdia patients.

### 3.6. Genetic Analysis of the Adia Cohort

In this last step, our study sought to identify gene variants in atypical diabetes patients (Adia cohort), using gene panel and WES.

The MODY gene panel analysis was first performed on the whole Adia cohort, without any pathological variants identified in the analyzed patients.

Then, a WES was conducted, revealing 61 gene variants in 34 patients, while 21 patients did not harbor any genetic variant for the targeted genes (Table 4). According to the databases, among the 61 detected variants, 28 were reported as benign/likely benign (Class I/II) variants, 28 as variants of uncertain significance (Class III), and five as likely pathogenic (Class IV). The *FOXP3* (c.970T>C) variant was the only probably pathological variant identified. While no assertion criteria were provided in ClinVar, the *FOXP3* gene displayed low-pathogenicity predictor scores (Polyphen2 [TOL], the REVEL score [0.564], and CADD [17,24]). By looking for our variants in the literature available on Pubmed, we found that one of our Adia patients carried a mutation in the *CEL* gene (c.1818delC) that was reported to cause MODY type 8 (MODY8) [28]. Furthermore, another patient exhibited two similar mutations for which no information was available in the literature: *CEL* (c.1810delG) and *CEL* (c.1819_1982del). For one of them, *CEL* (c.1819_1982del), the deletion induced the same frameshift (p.Val607fs) as the one reported to be a causative agent for MODY8, pleading in favor of a causative role when it occurs in an isolated manner. To our knowledge, his deletion has not yet been reported so far, but its interpretation is complicated, as it is added to a second frameshift deletion.

Moreover, the variants classified as “unknown” were neither found in ClinVar, SNP, and LOVD, nor in the published scientific literature, thus rendering confirmation of their potential pathogenicity complex. Consequently, the variants were classified according to the ACMG guidelines as “Class I”, “Class II”, or “Class III”. Among the 28 Class III variants, 24 were missense variants, two were splice site region variants in *STAT1* and *EIF2B1* genes, one was splice site donor variant in *SLC2A2* gene and the last one was frameshift deletions in *MAFA* gene. It is worth noting that all the identified variants were heterozygous. Of these variants, 24 were classified as deleterious by MutationTaster and CADD scores (>20). Notably, five missense Class III/IV variants in *PCBD1* (c.205G>A, p.Val69Met), *WFS1* (c.1124G>A, p.Arg375His), *SLC2A2* (c.1214T>G, p.Phe405Cys), *WFS1* (c.961A>G, p.Thr321Ala), and ZMPSTE24 (c.505A>C, p.Lys169Gln) genes demonstrated strong-pathogenicity predictions based on REVEL and CADD scores (>0.7, >24), being relatively or absolutely absent in the general population (gnomAD database).

All corresponding variants and their characteristics are listed in Table 4.

## 4. Discussion

Diabetes is a heterogeneous disease whose classification is constantly evolving. In pediatrics, T1D is the most common form of diabetes (±90%), resulting from insulin deficiency caused by autoimmune destruction of pancreatic β-islet cells. In 1–4% of pediatric diabetes patients, a genetic disorder is responsible for the disease, called monogenic diabetes [2]. In addition to having a different etiology, monogenic diabetes is known to be easier to control and, theoretically, less severe than T1D, both at diagnosis and during the course of the disease.

In our study, we demonstrated that T1D and MODY patients differed in terms of clinical disease manifestations (diabetic ketoacidosis), as well as glycemic parameters, including glycemia, HbA_1c_, CV, TIR, and total daily insulin dose, both upon diagnosis and during the disease course. These findings support the notion that monogenic diabetes is less severe than T1D. Furthermore, we demonstrated that patients with atypical diabetes, as represented by our Adia cohort, differed from patients with T1D and MODY, displaying intermediate values for the different parameters studied.

Then, we took it a step further and divided the Adia cohort according to the number of positive DIAMODIA criteria met: two, three, or at least four. Patients displayed intermediates values within these subsets, progressively bridging the gap between the T1D and MODY cohorts. These intermediate forms of diabetes further highlight the clinical heterogeneity of diabetes patients and complicate precise etiological diagnosis by revealing the overlap between the different forms of diabetes. In this sense, Bielka et al. published an article about how the definition of “double diabetes” (also known as “hybrid diabetes” or “type 1.5 diabetes”) has changed over the years. It generally describes individuals diagnosed with T1D who share the insulin resistance characteristic of metabolic syndrome [29]. Additionally, another article has highlighted the ambiguity between typical diabetes and monogenic diabetes. According to the authors of that article, the key feature that distinguish monogenic diabetes from T1D are autoantibody negativity, the presence of C-peptide, and lower HbA_1c_, particularly for *GCK*-MODY. The key features that distinguish MODY from T2D are younger age at diagnosis, lower BMI, and autosomal dominant history [4].

Additionally, our correlation analysis revealed that patients exhibiting more DIAMODIA criteria had glycemic parameters closer to treatment objectives. This finding aligns with our analysis of glycemic variability parameters. We demonstrated that MODY patients exhibited better diabetes management compared to Tdia patients. The DIA4+ patients, who have atypical diabetes and present with at least four positive DIAMODIA criteria, were closest to the MODY patients in terms of values. However, MODY patients are genetically heterogeneous and are treated differently than patients from others cohorts because only a small proportion of them are under insulin. Therefore, these two parameters may constitute major confounding factors.

Regarding the genetic analyses, the MODY gene panel failed to identify any known pathological variants in our patients with atypical diabetes. Notably, no genetic variants were found in some Adia patients who shared the same DIAMODIA characteristics compared to the majority of MODY patients. These Adia patients are represented in our MCA by a green # close to the blue dots. However, these patients could still carry variants of diabetes-related genes that have not yet been recognized.

However, one patient displayed a probably pathological variant in the *FOXP3* gene. The *FOXP3* gene is known to play a role in immunological tolerance, and a pathological variant can lead to autoimmune diseases such as T1D [30].

Furthermore, the DIAMODIA criteria facilitated the diagnosis of MODY8 in a patient who tested positive for atypical diabetes. This allowed the patient to undergo genetic testing, which revealed a mutation that had only been documented once before in the *CEL* (carboxyl ester lipase) gene. This gene encodes one of the major lipases produced in pancreatic acinar cells and secreted into pancreatic juice [28]. Researchers have demonstrated that frameshifts in the C-terminal region of the *CEL* gene can result in the production of a misfolded protein that aggregates and induces acinar cell apoptosis via endoplasmic reticulum stress and the unfolded protein response [31]. This case illustrates the possibility that other variants described in our article may also be responsible for certain subtypes of diabetes. This could be interpreted as evidence that characterization of other existing forms of monogenic diabetes may still be missing. In this regard, further molecular and trio analyses in the context of clinical studies are needed to assess the potential causal link between a given genetic variant and the corresponding subtype of diabetes.

Correspondingly, five missense Class III variants were noted to have a strong pathogenicity prediction according to the REVEL (>0.7) and CADD (>24) score algorithms, without any observation in the general population (gnomAD database). The *PCBD1* gene, which is among these variants, encodes an enzyme responsible for the homodimerization of the transcription factor hepatocyte nuclear factor 1. This transcription factor is known to cause MODY type 3 in the event of a mutation [32]. Another variant, the *WFS1* gene, encodes an ion channel in the endoplasmic reticulum, specifically in pancreatic β-cells. Wolfram syndrome (DIDMOAD: Diabetes insipidus, diabetes mellitus, optic atrophy, and deafness) is caused by pathological mutations of the *WFS1* gene [33]. The last two variants are solute carrier family 2 (facilitated glucose transporter), member 2 (SLC2A2), also known as glucose transporter 2 (GLUT2) [34], and a metallopeptidase (ZMPSTE24), which is involved in the processing of lamin A [35].

Moreover, we propose several recommendations for the implementation of the DIAMODIA criteria in clinical practice. We suggest using the DIAMODIA criteria to screen children initially diagnosed with type 1 diabetes (T1D) after the remission period, or in cases of prolonged remission (e.g., lasting more than one year). Patients meeting at least one strong criterion and one weak criterion should be considered as having atypical diabetes and may be referred for genetic testing after obtaining free and informed consent from the patient or their legal guardians.

However, the cost of genetic testing represents a major limitation in the diagnostic process. It should be noted that genetic testing may be available free of charge within the framework of research programs, as outlined in the ISPAD (International Society for Pediatric and Adolescent Diabetes) guidelines [7]. Once a genetic variant is identified, it must be interpreted according to ACMG guidelines and classified into one of five categories [23]. If the variant is classified as benign or likely benign, an alternative subtype of diabetes should be considered. Conversely, if the variant is pathogenic or likely pathogenic, there is sufficient evidence to conclude that it is causative of the disease.

When a variant of unknown significance is identified, or prior to performing predictive testing in asymptomatic children, referral to an expert center is recommended to avoid misdiagnosis and unnecessary testing, which may be harmful [7]. In contrast, and in accordance with ISPAD recommendations, all infants diagnosed with diabetes within the first six months of life should undergo immediate genetic testing, as T1D is extremely rare in this age group [7].

Our research has several limitations that must be mentioned. First, the retrospective nature of our study resulted in numerous missing values. The following islet autoantibodies were tested in the majority of our patients: glutamic acid decarboxylase 65 (GAD65) antibodies, insulinoma-associated antigen-2 (IA-2) antibodies, insulin antibodies and zinc transporter 8 (ZnT8) antibodies. However, due to the retrospective nature of the study, some patients were not tested for one or more of these autoantibodies. This could potentially lead to patient misclassification. Additional analyses regarding autoantibody testing results are reported in the Supplementary Materials. Then, the subgroups (DIA2, DIA3 and DIA4+) were small and therefore several direct comparisons did not reach statistical significance, even when the overall *p*-value was. In the same way, as CGM data were available only for a very small proportion of our cohort, many subgroup comparisons were exploratory and potentially unstable. In addition, it is important to remember that all CGM data comes from patients affiliated with a single clinical center. This may have reduced control over bias and limit generalizability. In addition, differences seen between MODY and others cohorts in terms of glycemic parameters were likely influenced by genetic heterogeneity and by a lower proportion of patients under insulin in the MODY group. These two parameters, for which any adjusted or stratified analyses were realized, remain potential contributors to the gradient observed. However, to our knowledge, there is no other study describing an overlap in the glycemic parameters of patients with different types of diabetes. Based on our initial exploratory results, we present a new methodological approach that could be replicated by other researchers in the future. Furthermore, the DIAMODIA criteria were developed by our group and, to our knowledge, have not yet been validated by another team. To date, there is therefore no external validation in independent populations. IDAA_1c_ ≤ 9 and GTAA_1c_ ≤ 4.5, two strong DIAMODIA criteria, are tightly linked to glycemic control, which is then used as an outcome. This creates a risk of circular reasoning. Additionally, our study did not include trio analysis of Adia family members for genetic sequencing and no functional validation of the variants has been performed. This limited our ability to validate variants observed in our Adia cohort, particularly those in the *FOXP3* and *CEL* genes. That is why their interpretation remains hypothesis-oriented. However, there is a high likelihood that a fraction of the Adia cohort could be, in fact, included in the MODY cohort. Finally, since all our patients were pediatric and under 18 years old, we were unable to identify clinical features that would occur after several decades of diabetes.

## 5. Conclusions

In conclusion, the GENEPEDIAB study showed an overlap among the different types of diabetes, whether in terms of glycemic variability or according to the DIAMODIA criteria. We have highlighted that, in our specific cohort of pediatric patients, children with atypical diabetes displayed intermediates clinical phenotype between T1D and MODY patients. Within the Adia sub-cohorts, we observed a gradient, with patients exhibiting fewer positive DIAMODIA criteria being closer to T1D patients and those exhibiting more positive criteria being closer to MODY patients. External validity of our study is mitigated due to the weaknesses described in the limitations paragraph. Therefore, other investigations such as cross-validation studies will be needed in order to assess whether our results can be generalized to patients other than those included in our study. Additionally, through genetic analyses, we identified one MODY8 in our cohort of patients presenting atypical diabetes according to our DIAMODIA criteria. It is likely that in the future a causal link will be established between some variants found in the Adia cohort and subtypes of diabetes that have not yet been described. These results provide new insights into the clinical presentation of pediatric diabetes, offering prospects for improved diagnosis and care for diabetic patients.

## Figures and Tables

**Table 1:** List of genes involved in monogenic, neonatal, and very rare forms of diabetes based on the EXETER genes list.

*ABCC8*	*COQ9*	*FOXP3*	*IER3IP1*	*MNX1*	*PLIN1*	*STAT3*
*AGPAT2*	*CP*	*GATA4*	*IL2RA*	*MTTL1*	*POLD1*	*STAT5B*
*AIRE*	*CTLA4*	*GATA6*	*INS*	*NEUROD1*	*PPARG*	*TNFAIP3*
*AKT2*	*DCAF17*	*GCK*	*INSR*	*NEUROG3*	*PPP1R15B*	*TRMT10A*
*APPL1*	*DEXI*	*GLIS3*	*ITCH*	*NKX2*	*PTF1A*	*WFS1*
*BLK*	*DNAJC3*	*GLUD1*	*JAK1*	*NKX2-2*	*RFX6*	*ZBTB20*
*BSCL2*	*DOCK8*	*HADH*	*KCNJ11*	*PAX4*	*SIRT1*	*ZFP57*
*CDKN1C*	*DUT*	*HNF1A*	*KLF11*	*PAX6*	*SLC19A2*	
*CEL*	*DYRK1B*	*HNF1B*	*LMNA*	*PCBD1*	*SLC29A3*	
*CISD2*	*EIF2AK3*	*HNF4A*	*LPL*	*PDX1*	*SLC2A2*	
*COQ2*	*EIF2S3*	*ICOS*	*LRBA*	*PIK3R1*	*STAT1*	

**Table 2:** Clinical data of Tdia, Adia, and MODY cohorts.

	Tdia (*n* = 337)	Adia (*n* = 75)	MODY (*n* = 34)	*p*-Value Tdia vs. Adia	*p*-Value Adia vs. MODY	*p*-Value Global
**Patient data**						
Gender—Girls (%)	51.6	42.7	41.2	0.26 ^‡^	0.84 ^‡^	0.31 ^‡^
**Neonatal history**						
Birth weight (kg)	3.3 ± 0.6	3.4 ± 0.5	3.2 ± 0.6	0.81 ^¥^	0.07 ^¥^	0.21 ^‡^
Birth height (cm)	49.81 ± 2.8	50.68 ± 2.8	49.47 ± 3.9	0.04 * ^¥^	0.10 ^¥^	0.21
Pre-term (%)	8.3	1.9	7.7	0.10 ^‡^	0.13 ^‡^	0.25 ^‡^
Term (%)	83.7	90.7	84.6	0.20 ^‡^	0.26 ^‡^	0.43 ^‡^
Post-term (%)	8.0	7.4	7.7	0.91 ^‡^	0.83 ^‡^	0.98 ^‡^
Neonatal hypoglycemia (%)	2.9	3.9	25.9	0.75 ^‡^	0.002 ** ^‡^	<0.001 *** ^‡^
Gestational diabetes (%)	2.9	3.6	14.3	0.75 ^‡^	0.005 ** ^‡^	<0.001 *** ^‡^
**Diabetes diagnosis**						
Age (years)	8.18 ± 3.8	8.24 ± 3.8	6.98 ± 4.9	0.91 ^¥^	0.19 ^¥^	0.22
Height (SDS)	0.03 ± 1.1	0.12 ± 1.4	−0.31 ± 1.3	0.64 ^¥^	0.69 ^¥^	0.23
Weight (SDS)	−0.49 ± 1.2	−0.30 ± 1.3	−0.07 ± 1.2	0.32 ^¥^	0.43 ^¥^	0.14
BMI (SDS)	−0.82 ± 1.6	−0.61 ± 1.8	0.07 ± 1.6	0.51 ^¥^	0.008 * ^¥^	0.013 *
Fasting blood glucose (mg/dL)	478.95 ± 215.2	405.28 ± 209.9	203.38 ± 194.7	0.02 * ^¥^	<0.001 *** ^¥^	<0.001 ***
HbA_1c_ (%)	11.47 ± 2.2	10.88 ± 2.4	7.14 ± 2.2	0.09 ^¥^	<0.001 *** ^¥^	<0.001 ***
Diabetic ketoacidosis (%)	38.1	28.4	12.1	0.17 ^‡^	0.07 ^‡^	0.005 ** ^‡^
Auto-immune disease (%)	14.6	6.8	2.9	0.17 ^‡^	0.36 ^‡^	0.07 ^‡^
**Glycemic parameters**						
HbA_1c_ (%)	7.49 ± 1.1	6.52 ± 0.6	6.18 ± 0.9	<0.001 *** ^¥^	<0.02 * ^¥^	<0.001 ***
TIR_70–180_ (%)	43.12 ± 10.9	53.09 ± 13.8	76.70 ± 17.3	<0.001 *** ^¥^	<0.001 *** ^¥^	<0.001 *** ^¥^
IDAA_1c_ score	11.34 ± 1.4	9.59 ± 1.5	7.09 ± 1.9	<0.001 *** ^¥^	<0.001 *** ^¥^	<0.001 ***
GTAA_1c_ score	6.25 ± 1.0	4.93 ± 0.9	3.79 ± 1.3	<0.001 *** ^¥^	<0.001 *** ^¥^	<0.001 ***
Glycemic parameters after CGM retrieval	*n* = 21	*n* = 27	*n* = 2			
Mean (mg/dL)	186.81 ± 41.49	165.17 ± 34.28	131.47 ± 23.49	0.23	0.41	0.07
Median (mg/dL)	173.88 ± 43.71	155.17 ± 34.36	129.00 ± 24.04	0.26	0.63	0.14
Min (mg/dL)	41.90 ± 4.24	43.04 ± 5.38	58.50 ± 24.75	0.81	0.004 **	0.003 **
Max (mg/dL)	469.81 ± 50.13	464.26 ± 68.37	256.50 ± 31.82	0.94	<0.001 ***	<0.001 ***
GMI (%)	8.06 ± 1.43	7.40 ± 1.20	6.16 ± 0.81	0.23	0.41	0.07
CV	0.49 ± 0.07	0.47 ± 0.09	0.21 ± 0.12	0.69	<0.001 ***	<0.001 ***
TIR_70–180_	0.47 ± 0.13	0.51 ± 0.14	0.93 ± 0.04	0.26	0.001 **	<0.001 ***
TIR_70–140_	0.31 ± 0.10	0.37 ± 0.15	0.55 ± 0.30	0.30	0.23	0.05
TIR_60–140_	0.35 ± 0.12	0.42 ± 0.15	0.57 ± 0.33	0.28	0.37	0.08
TBR_<70_	0.09 ± 0.05	0.11 ± 0.07	0.03 ± 0.04	0.77	0.25	0.26
TBR_<54_	0.01 ± 0.02	0.02 ± 0.03	0.00 ± 0.00	0.50	0.41	0.31
TAR_>180_	0.45 ± 0.15	0.38 ± 0.14	0.05 ± 0.01	0.19	0.02 *	0.002 **
TAR_>250_	0.24 ± 0.15	0.17 ± 0.13	0.00 ± 0.00	0.24	0.22	0.04 *
Hypoglycemia (/day)	0.54 ± 0.37	0.66 ± 0.46	0.26 ± 0.36	0.62	0.40	0.34
PHH (/day)	0.12 ± 0.10	0.22 ± 0.25	0.05 ± 0.06	0.31	0.48	0.23
PHH/Hypo ratio	0.28 ± 0.22	0.29 ± 0.26	0.09 ± 0.12	0.9	0.53	0.54
PHH duration (min)	381.44 ± 203.89	263.89 ± 197.21	8.75 ± 12.37	0.13	0.002 **	<0.001 ***
**Chronic treatment**						
Insulin treatment (%)	100	96	26.5	N/A	N/A	N/A
Antidiabetic drugs (%)						
Biguanide (%)	3.3	6.7	8.8	N/A	N/A	N/A
Sulfonylureas (%)	0	0	5.8	N/A	N/A	N/A
Glinides (%)	0	0	2.9	N/A	N/A	N/A
**DIAMODIA criteria**						
	**Tdia** **(*n* = 337)**	**Adia** **(*n* = 75)**	**MODY** **(*n* = 34)**	***p*-value**		
**Strong criteria**						
Absence of anti-islet antibodies (%)	0.6	40.6	100	1 × 10^−12^ *** ^‡^		
IDAA_1c_ < 9 (%)	0.6	50.0	80.0	1 × 10^−15^ *** ^‡^		
GTAA_1c_ < 4.5 (%)	0.9	38.9	81.5	1 × 10^−15^ *** ^‡^		
Age at diagnosis < 6 months (%)	0	0	8.8	1 × 10^−12^ *** ^‡^		
**Weak criteria**						
First-degree relative with diabetes (%)	41.0	56.8	85.3	1 × 10^−6^ *** ^‡^		
C-peptide secretion positive (%)	23.9	59.4	90.6	1 × 10^−9^ *** ^‡^		
Extra-pancreatic manifestations (%)	8.5	13.3	38.7	2 × 10^−6^ *** ^‡^		
Absence of ketoacidosis at diagnosis (%)	61.8	73.3	87.9	0.005 ** ^‡^		
History of neonatal hypoglycemia (%)	2.9	2.7	25.9	1 × 10^−6^ *** ^‡^		

The plus-minus values are means ± SD. Percentages may not total 100 due to rounding. Differences between the three cohorts were considered significant when the *p*-value was under 0.05. The level of significance is represented as follows: nonsignificant, *p* < 0.05 (*), *p* < 0.01 (**), *p* < 0.001 (***). ^¥^ Kruskal–Wallis test; ^‡^ Chi-square. Total: All diabetic patients in our study; Tdia: Cohort of T1D patients; Adia: Cohort of patients with atypical diabetes; MODY: Cohort of patients with maturity-onset diabetes of the young; BMI: Body mass index; SDS: Standard deviation score; HbA_1c_: Glycated hemoglobin (%); IDAA_1c_: Insulin-dose adjusted A1c; GTAA_1c_: Glycemic target-adjusted HbA_1c_; Mean: Mean glycemia (mg/dL); Median: Median glycemia (mg/dL); Min: Minimum glycemia (mg/dL); Max: Maximum glycemia (mg/dL); GMI: Glucose management indicator (%); CV: Coefficient of variation; TIR_70–180_: Time in range (70–180 mg/dL); TIR_70–140_: Time in range (70–140 mg/dL); TIR_60–140_: Time in range (60–140 mg/dL); TBR_<70_: Time below the range (<70 mg/dL); TBR_<54_: Time below the range (<54 mg/dL); TAR_>180_: Time above the range (>180 mg/dL); TAR_>250_: Time above the range (>250 mg/dL); Hypoglycemia (/day): Number of episodes of glycemia < 54 mg/dL per day; PHH (/day): Post-hypoglycemia hyperglycemia/day; PHH/Hypo ratio: Post-hypoglycemia hyperglycemia/hypoglycemia ratio; PHH duration: Post-hypoglycemia hyperglycemia mean duration (minutes); DIAMODIA criteria: DIAgnose Monogenic DIAbetes criteria. For the DIAMODIA criteria, percentages may not total 100 due to rounding. N/A: not acquired.

**Table 3:** Clinical data of Tdia, Adia (DIA2, DIA3, and DIA4+ subgroups), and MODY cohorts.

Cohorts	Tdia *n* = 337	DIA2 *n* = 17	T1D vs. DIA2 *p*-Value	DIA3 *n* = 26	DIA2 vs. DIA3 *p*-Value	DIA4+ *n* = 28	DIA3 vs. DIA4+ *p*-Value	MODY *n* = 34	DIA4+ vs. MODY *p*-Value	Global *p*-Value
**Patient data**										
**Gender—Girl (%)**	51.63	35.29	0.18	42.31	0.65	53.57	0.41	41.18	0.33	0.45
**Neonatal history**										
**Birth weight (kg)**	3.35 ± 0.62	3.34 ± 0.33	0.99	3.36 ± 0.64	0.99	3.32 ± 0.56	0.95	3.14 ± 0.57	0.88	0.42
**Birth height (cm)**	49.81 ± 2.76	51.33 ± 1.63	0.97	50.68 ± 2.03	0.99	50.15 ± 3.76	0.99	49.48 ± 3.87	0.98	0.90
**Term**										
**Pre-term (%)**	8.33	0	0.21	0	1	5.0	0.33	7.69	0.40	0.32
**Term (%)**	83.70	90.91	0.61	89.47	0.98	90.0	0.92	84.62	0.64	0.88
**Post-term (%)**	7.97	9.09	0.58	10.53	0.98	5.0	0.26	7.69	0.40	0.82
**Neonatal hypoglycemia (%)**	2.91	0	0.47	5.88	0.24	3.57	0.51	21.21	0.046 *	<0.001 ***
**Gestational diabetes (%)**	2.89	9.09	0.05	0	0.07	5.0	0.33	14.29	0.14	0.005 **
**Diabetes diagnosis**										
**Age (years)**	8.17 ± 3.83	6.83 ± 3.34	0.47	8.16 ± 3.52	0.87	9.11 ± 4.18	0.11	6.98 ± 4.95	0.12	0.01 *
**Height (SDS)**	0.03 ± 1.08	0 ± 1.06	0.61	0.51 ± 0.93	0.99	−0.18 ± 1.73	0.71	−0.31 ± 1.27	0.98	0.18
**Weight (SDS)**	−0.49 ± 1.25	−0.8 ± 1.37	0.31	−0.48 ± 1.22	0.99	0.03 ± 1.34	0.96	−0.07 ± 1.23	0.97	0.02 *
**BMI (SDS)**	−0.82 ± 1.57	−1.49 ± 1.77	0.57	−1.15 ± 1.62	0.95	0.16 ± 1.66	0.01 *	0.07 ± 1.59	0.99	<0.001 ***
**Fasting blood glucose (mg/dL)**	478.95 ± 215.16	416.86 ± 144.31	0.58	422.04 ± 126.22	0.59	336.52 ± 172.96	0.63	203.38 ± 194.70	0.04 *	<0.001 ***
HbA_1c_ **(%)**	11.47 ± 2.20	11.74 ± 1.58	0.59	11.41 ± 2.10	0.61	9.60 ± 2.34	0.008 **	7.14 ± 2.23	0.04 *	<0.001 ***
**Diabetic ketoacidosis (%)**	38.15	47.06	0.47	26.92	0.18	7.14	0.05	11.76	0.54	<0.001 ***
**Autoimmune disease (%)**	14.63	5.88	0.32	7.69	0.82	3.70	0.51	2.94	0.89	0.11
**Glycemic parameters**										
HbA_1c_ **(%)**	7.49 ± 1.1	6.80 ± 0.62	<0.001 ***	6.40 ± 0.60	0.04 *	6.40 ± 0.65	1	6.18 ± 0.9	<0.001 ***	<0.001 ***
**TIR_70–180_ (%)**	43.12 ± 10.9	52.20 ± 9.73	0.33	48.20 ± 11.18	0.98	58.80 ± 17.32	0.007 **	76.70 ± 17.3	<0.001 ***	<0.001 ***
**IDAA_1c_ score**	11.34 ± 1.4	9.90 ± 1.59	0.04 *	9.40 ± 1.04	0.11	9.40 ± 1.86	0.91	7.09 ± 1.9	<0.001 ***	<0.001 ***
**GTAA_1c_ score**	6.25 ± 1.0	5.20 ± 0.76	0.84	4.90 ± 0.82	0.18	4.70 ± 0.97	0.03 *	3.79 ± 1.3	<0.001 ***	<0.001 ***
**Glycemic parameters after CGM retrieval**	**Tdia** ***n* = 21**	**DIA2** ***n* = 13**	**T1D vs. DIA2** ***p*-value**	**DIA3** ***n* = 9**	**DIA2 vs. DIA3** ***p*-value**	**DIA4+** ***n* = 5**	**DIA3 vs. DIA4+** ***p*-value**	**MODY** ***n* = 2**	**DIA4+ vs. MODY** ***p*-value**	**Global *p*-value**
HbA_1c_ **(%)**	8.06 ± 1.43	7.51 ± 1.43		7.44 ± 1.02		7.05 ± 1.10		6.16 ± 0.81		
**Mean (mg/dL)**	186.81 ± 41.49	168.86 ± 40.09	0.79	178.14 ± 20.92	0.99	150.90 ± 31.13	0.99	131.47 ± 23.49	0.94	0.25
**Median (mg/dL)**	173.88 ± 43.71	157.73 ± 40.00	0.81	168.33 ± 23.03	0.99	149.00 ± 28.19	0.99	129.00 ± 24.04	0.96	0.41
**Min (mg/dL)**	41.90 ± 4.24	42.18 ± 4.38	0.99	41.17 ± 2.86	0.99	47.75 ± 9.18	0.52	58.50 ± 24.75	0.26	0.006 **
**Max (mg/dL)**	469.81 ± 50.13	477.55 ± 41.01	0.99	492.67 ± 17.96	0.99	399.50 ± 131.22	0.16	256.50 ± 31.82	0.04 *	<0.001 ***
**GMI (%)**	8.06 ± 1.43	7.51 ± 1.43	0.79	7.87 ± 0.73	0.99	7.05 ± 1.10	0.99	6.16 ± 0.81	0.94	0.25
**CV**	0.49 ± 0.07	0.48 ± 0.05	0.99	0.50 ± 0.04	0.97	0.36 ± 0.15	0.02 *	0.21 ± 0.12	0.13	<0.001 ***
**TIR_70–180_**	0.47 ± 0.13	0.50 ± 0.13	0.78	0.44 ± 0.06	0.97	0.62 ± 0.21	0.23	0.93 ± 0.04	0.18	<0.001 ***
**TIR_70–140_**	0.31 ± 0.10	0.36 ± 0.09	0.84	0.29 ± 0.05	0.98	0.49 ± 0.31	0.29	0.55 ± 0.30	0.99	0.04 *
**TIR_60–140_**	0.35 ± 0.12	0.42 ± 0.10	0.77	0.34 ± 0.06	0.99	0.51 ± 0.30	0.62	0.57 ± 0.33	0.99	0.14
**TBR_<70_**	0.09 ± 0.05	0.11 ± 0.05	0.95	0.11 ± 0.03	0.82	0.07 ± 0.08	0.04 *	0.03 ± 0.04	0.99	0.03 *
**TBR_<54_**	0.01 ± 0.02	0.02 ± 0.03	0.97	0.03 ± 0.02	0.64	0.01 ± 0.01	0.28	0.00 ± 0.00	0.99	0.16
**TAR_>180_**	0.45 ± 0.15	0.39 ± 0.14	0.69	0.45 ± 0.08	0.99	0.31 ± 0.18	0.90	0.05 ± 0.01	0.27	0.01 *
**TAR_>250_**	0.24 ± 0.15	0.17 ± 0.15	0.74	0.24 ± 0.09	0.99	0.11 ± 0.08	0.88	0.00 ± 0.00	0.87	0.12
**Hypoglycemia (/day)**	0.54 ± 0.37	0.72 ± 0.52	0.76	0.74 ± 0.37	0.99	0.25 ± 0.26	0.20	0.26 ± 0.36	0.99	0.12
**PHH (/day)**	0.12 ± 0.10	0.16 ± 0.15	0.97	0.40 ± 0.36	0.29	0.08 ± 0.10	0.19	0.05 ± 0.06	0.99	0.07
**PHH/Hypo ratio**	0.28 ± 0.22	0.20 ± 0.14	0.91	0.45 ± 0.33	0.58	0.28 ± 0.35	0.98	0.09 ± 0.12	0.86	0.49
**PHH duration (min)**	381.44 ± 203.89	246.42 ± 173.25	0.03 *	425.08 ± 227.20	0.06	121.27 ± 107.29	<0.001 ***	8.75 ± 12.37	0.04 *	<0.001 *
**Severe hypoglycemia**										
**History of SH, yes**	12/21	7/22	0.09	2/28	0.02 *	1/28	0.55	0/34	0.27	<0.001 ***
**Chronic treatment**										
**Insulin treatment (%)**	100	100	1	100	1	89.29	0.09	26.47	<0.001 ***	<0.001 ***
**Antidiabetic drugs (%)**	3.56	0	0.42	0	1	21.43	0.01 *	17.65	0.71	<0.001 ***
**Biguanide (%)**	3.26	0	N/A	0	N/A	19.23	N/A	8.82	N/A	N/A
**SGLT2 inhibitor (%)**	0	0	N/A	0	N/A	3.57	N/A	0	N/A	N/A
**Sulfonylureas (%)**	0.30	0	N/A	0	N/A	0	N/A	5.88	N/A	N/A
**Incretines (%)**	0	0	N/A	0	N/A	3.57	N/A	0	N/A	N/A
**Glinides (%)**	0	0	N/A	0	N/A	0	N/A	2.94	N/A	N/A
**DIAMODIA criteria**										
**Strong criteria**										
**Absence of anti-islet antibodies (%)**	0.62	41.18	<0.001 ***	33.33	0.66	50.0	0.25	100	<0.001 ***	<0.001 ***
**IDAA_1c_ < 9 (%)**	1.51	29.41	<0.001 ***	60.0	0.04 *	70.37	0.44	82.35	0.31	<0.001 ***
**GTAA_1c_ < 4.5 (%)**	1.20	11.76	<0.001 ***	41.67	0.03 *	59.26	0.18	82.14	0.13	<0.001 ***
**Age at diagnosis < 6 months (%)**	0	0	1	0	1	0	1	8.82	0.11	<0.001 ***
**Weak criteria**										
**First-degree relative with diabetes (%)**	41.02	25.0	0.15	65.38	0.007 **	71.43	0.63	85.29	0.18	<0.001 ***
**C-peptide secretion positive (%)**	23.86	31.25	0.59	43.48	0.39	92.59	<0.001 *	90.62	0.81	<0.001 ***
**Extra-pancreatic manifestations (%)**	8.46	12.50	0.65	7.69	0.65	21.43	0.16	38.71	0.15	<0.001 ***
**Absence of ketoacidosis at diagnosis (%)**	60.55	52.94	0.53	73.08	0.18	92.86	0.05	88.24	0.54	<0.001 ***
**History of neonatal hypoglycemia (%)**	2.44	0	0.44	5.88	0.24	3.57	0.51	21.21	0.046 *	<0.001 ***

The plus-minus values are means ± SD. Percentages may not total 100 due to rounding. Differences between the three cohorts were considered significant when the *p*-value was 0.05. The level of significance is represented as follows: non-significant, *p* < 0.05 (*), *p* < 0.01 (**), *p* < 0.001 (***). Total: All diabetic patients in our study; Tdia: Type 1 diabetes; Adia: Atypical diabetes; MODY: Maturity-onset diabetes of the young; DIAMODIA: DIAgnose Monogenic DIAbetes; DIA2: Cohort of patients with two positive DIAMODIA criteria; DIA3: Cohort of patients with three positive DIAMODIA criteria; DIA4+: Cohort of patients with four or more DIAMODIA criteria; BMI: Body mass index; SDS: Standard deviation score; HbA_1c_: Glycated hemoglobin (%); IDAA_1c_: Insulin-dose adjusted A1c; GTAA_1c_: Glycemic target-adjusted HbA_1c_; Mean: Mean glycemia (mg/dL); Median: Median glycemia (mg/dL); Min: Minimum glycemia (mg/dL); Max: Maximum glycemia (mg/dL); GMI: Glucose management indicator (%); CV: Coefficient of variation; TIR_70–180_: Time in range (70–180 mg/dL); TIR_70–140_: Time in range (70–140 mg/dL); TIR_60–140_: Time in range (60–140 mg/dL); TBR_<70_: Time below the range (<70 mg/dL); TBR_<54_: Time below the range (<54 mg/dL); TAR_>180_: Time above the range (>180 mg/dL); TAR_>250_: Time above the range (>250 mg/dL); Hypoglycemia (/day): Number of episodes of glycemia < 54 mg/dL per day; PHH (/day), Post-hypoglycemia hyperglycemia/day; PHH/Hypo ratio, Post-hypoglycemia hyperglycemia/hypoglycemia ratio; PHH duration: Post-hypoglycemia hyperglycemia mean duration (minutes); SGLT2: Sodium-glucose co-transporter 2; N/A: not acquired.

**Table 4:** Variant identification.

*N*°	Gene	Variant	AA Change	Consensus	Allele Counts	Homozy-Gotes	Function
**Class I/II**							
4	INSR	c.2542+3A>G	N/A	300	14,997	164	Splice site region_SNV
28	DOCK8	c.3208A>G	p.Asn1070Asp	6	10,001	476	Nonsynonymous_SNV
32	DOCK8	c.3208A>G	p.Asn1070Asp	6	10,001	476	Nonsynonymous_SNV
11	AGPAT2	c.475C>T	p.Arg159Cys	10	8109	36	Nonsynonymous_SNV
13	KCNJ11	c.1154C>G	p.Ser385Cys	6	6323	54	Nonsynonymous_SNV
17	COQ9	c.835G>A	p.Asp279Asn	8	6087	16	Nonsynonymous_SNV
22	WFS1	c.577A>C	p.Lys193Gln	9	5981	29	Nonsynonymous_SNV
32	CEL	c.362T>G	p.Leu121Arg	8	5128	132	Nonsynonymous_SNV
18	CEL	c.362T>G	p.Leu121Arg	8	5128	132	Nonsynonymous_SNV
34	CEL	c.362T>G	p.Leu121Arg	8	5128	132	Nonsynonymous_SNV
15	RFX6	c.1327C>T	p.His443Tyr	301	3993	80	Nonsynonymous_SNV
6	CEL	c.16C>T	p.Arg6Cys	5	3166	20	Nonsynonymous_SNV
32	CEL	c.16C>T	p.Arg6Cys	5	3166	20	Nonsynonymous_SNV
18	CEL	c.16C>T	p.Arg6Cys	5	3166	20	Nonsynonymous_SNV
7	DOCK8	c.452G>A	p.Arg151Gln	5	2846	9	Nonsynonymous_SNV
28	WFS1	c.2335G>A	p.Val779Met	9	2444	16	Nonsynonymous_SNV
2	AKT2	c.960+3G>A	N/A	300	1664	16	Splice site region_SNV
10	RFX6	c.2176C>G	p.Arg726Gly	6	1337	8	Nonsynonymous_SNV
32	DNAJC3	c.1060G>C	p.Glu354Gln	6	1251	11	Nonsynonymous_SNV
18	HNF4A	c.648+4A>G	N/A	300	1214	18	Splice site region_SNV
5	PIK3R1	c.427+5C>A	N/A	300	904	0	Splice site region_SNV
21	CEL	c.1810delG	p.Ala604fs	400	711	67	Frameshift deletion
33	CEL	c.1810delG	p.Ala604fs	400	711	67	Frameshift deletion
27	CEL	c.2181delT	p.Val728fs	400	599	6	Frameshift deletion
9	ABCC8	c.4629G>T	p.Lys1543Asn	7	373	1	Nonsynonymous_SNV
32	TRMT10A	c.665C>T	p.Ala222Val	11	245	0	Nonsynonymous_SNV
4	CNOT1	c.2282A>G	p.Asn761Ser	5	164	1	Nonsynonymous_SNV
15	GATA6	c.592C>G	p.Leu198Val	11	141	0	Nonsynonymous_SNV
**Class III**							
26	APPL1	c.2018C>G	p.Ser673Cys	10	1461	2	Nonsynonymous_SNV
23	POLD1	c.961G>A	p.Gly321Ser	8	697	1	Nonsynonymous_SNV
3	WFS1	c.1124G>A	p.Arg375His	16	538	0	Nonsynonymous_SNV
23	PTF1A	c.960C>A	p.Asn320Lys	12	381	1	Nonsynonymous_SNV
1	PCBD1	c.205G>A	p.Val69Met	17	335	1	Nonsynonymous_SNV
29	HNF4A	c.635C>T	p.Pro212Leu	13	213	0	Nonsynonymous_SNV
14	LRBA	c.109C>T	p.Pro37Ser	11	155	0	Nonsynonymous_SNV
2	LRBA	c.7880C>T	p.Thr2627Ile	10	89	0	Nonsynonymous_SNV
20	SLC2A2	c.496+1G>T	N/A	303	84	0	Splice site donor_SNV
3	STAT1	c.1632+5G>A	N/A	300	76	0	Splice site region_SNV
32	COQ9	c.143A>G	p.Asp48Gly	7	66	0	Nonsynonymous_SNV
19	DOCK8	c.4283A>T	p.Asn1428Ile	13	35	1	Nonsynonymous_SNV
30	WFS1	c.2495G>A	p.Arg832His	14	32	0	Nonsynonymous_SNV
24	EIF2B1	c.369G>A	p.Ala123Ala	300 *	24	0	Splice site region_SNV
1	LRBA	c.2480G>A	p.Arg827Gln	9	20	0	Nonsynonymous_SNV
8	LRBA	c.188G>C	p.Arg63Thr	11	9	0	Nonsynonymous_SNV
21	GLIS3	c.1066G>A	p.Gly356Ser	8	9	0	Nonsynonymous_SNV
33	GLIS3	c.1066G>A	p.Gly356Ser	8	9	0	Nonsynonymous_SNV
31	LRBA	c.274A>G	p.Ile92Val	6	8	0	Nonsynonymous_SNV
12	EIF2AK3	c.2134A>G	p.Ile712Val	5	7	0	Nonsynonymous_SNV
16	ZMPSTE24	c.505A>C	p.Lys169Gln	18	7	0	Nonsynonymous_SNV
25	MAFA	c.282delC	p.Pro96fs	400	6	0	Frameshift deletion
23	CNOT1	c.4336C>T	p.Arg1446Cys	14	5	0	Nonsynonymous_SNV
3	MNX1	c.434G>A	p.Gly145Asp	6	4	0	Nonsynonymous_SNV
24	PPARG	c.742G>A	p.Asp248Asn	7	4	0	Nonsynonymous_SNV
29	ABCC8	c.2126C>G	p.Thr709Arg	10	N/A	N/A	Nonsynonymous_SNV
3	KCNJ11	c.689T>C	p.Val230Ala	11	N/A	N/A	Nonsynonymous_SNV
6	SLC2A2	c.1214T>G	p.Phe405Cys	15	N/A	N/A	Nonsynonymous_SNV
**Class IV**							
33	CEL	c.1819_1982del	p.Val607fs	400	670	31	Frameshift deletion
21	CEL	c.1819_1982del	p.Val607fs	400	670	31	Frameshift deletion
16	CEL	c.1818delC	p.Val607fs	400	29	0	Frameshift deletion
14	WFS1	c.961A>G	p.Thr321Ala	19	11	0	Nonsynonymous_SNV
30	FOXP3	c.970T>C	p.Phe324Leu	8	2	2	Nonsynonymous_SNV

For each class, the detected gene variants and the corresponding amino acid (AA) changes are shown. The variants are arranged from the highest to the lowest population allele count and included their homozygosity rates. Consensus scores of 1–20 reflect the number of Highlander tools that predict the variant is damaging. Scores above 300 indicate splice site mutations, and scores above 400 indicate loss-of-function mutations. ‘*’ refers to mutations with a strong impact on splicing or on LOF tolerance. Numbers from one to thirty-four refer to the patient who bears the variant and is represented in the MCA (Figure 2). LOF: loss-of-function; SNV = single-nucleotide variant; N/A: not acquired.

## Data Availability

The data that support the findings of this study are available from the corresponding author upon reasonable request due to ethical restrictions.

## Supplementary Materials

The following supporting information can be downloaded at: https://www.mdpi.com/article/10.3390/cells15050484/s1. Table S1: Description of auto-antibody testing among T1D, Adia and MODY cohorts. Table S2: Description of auto-antibody testing among T1D, DIA2, DIA3, DIA4+ and MODY cohorts.

## Abbreviations

The following abbreviations are used in this manuscript:

ACMG	American College of Medical Genetics and Genomics
BMI	Body Mass Index
CADD	Combined Annotation-Dependent Depletion
CGM	Continuous Glucose Monitoring
CUSL	Cliniques universitaires Saint-Luc
CV	Coefficient of Variation
DIAMODIA	DIAgnose MOnogenic DIAbetes
DNA	Deoxyribonucleic Acid
GENEPEDIAB	GENEtic forms of PEDiatric DIABetes
GTAA_1c_ score	Glycemic Target-Adjusted HbA1c
HbA_1c_	Glycated Hemoglobin
IDAA_1c_ score	Insulin-Dose Adjusted A1c
ISPAD	International Society for Pediatric and Adolescent Diabetes
MCA	Multiple Correspondence Analysis
MLPA	Multiplex Ligation-dependent Probe Amplification
MODY	Maturity-Onset Diabetes of the Young
MODY 8	Maturity-Onset Diabetes of the Young Type 8
OGTT	Oral Glucose Tolerance Test
PHH	Post-Hypoglycemic Hyperglycemia
REVEL	Rare Exome Variant Ensemble Learner
SD	Standard Deviation
T1D	Type 1 Diabetes
T2D	Type 2 Diabetes
TAR	Time Above Range
TBR	Time Below Range
TIR	Time In Range
WES	Whole Exome Sequencing
