# Supplementary material for: Clinical Characterization of Atypical Diabetes: Insights from the GENEPEDIAB Study into the Spectrum Between Type 1 and Monogenic Diabetes
Source: Cells. 2026 Mar 7;15(5):484. doi: 10.3390/cells15050484 (PMC12984146; doi:10.3390/cells15050484)
Supplement: Supplementary file 1 [file cells-15-00484-s001.zip › cells-4117309-supplementary.pdf]

Supplementary Table S1: Description of auto-antibody testing among T1D, Adia and MODY cohorts.

	T1D	Adia	MODY
**Antibodies Tested**			
- **Yes**	325 (96.4%)	64 (85.3%)	25 (73.5%)
- **No**	1 (0.3%)	1 (1.3%)	9 (26.5%)
- **NA**	11 (3.4%)	10 (13.3%)	0 (0%)
**AB (All)**			
- **Present**	322 (95.5%)	38 (50.7%)	0 (0%)
- **Absent**	2 (0.6%)	26 (34.7%)	25 (73.5%)
- **NA**	13 (3.9%)	11 (14.7%)	9 (26.5%)
**AAB-GAD65**			
- **Present**	246 (73.0%)	26 (34.7%)	0 (0%)
- **Absent**	82 (24.3%)	49 (65.3%)	25 (73.5%)
- **NA**	9 (2.7%)	0 (0%)	9 (26.5%)
**AAB-IA2**			
- **Present**	239 (70.9%)	26 (34.7%)	0 (0%)
- **Absent**	89 (26.4%)	49 (65.3%)	25 (73.5%)
- **NA**	9 (2.7%)	0 (0%)	9 (26.5%)
**AAB-Ins**			
- **Present**	87 (25.8%)	13 (17.3%)	0 (0%)
- **Absent**	241 (71.5%)	62 (82.7%)	25 (73.5%)
- **NA**	9 (2.7%)	0 (0%)	9 (26.5%)
**AAB-Znt8**			
- **Present**	0 (0%)	1 (1.3%)	0 (0%)
- **Absent**	328 (97.3%)	74 (98.7%)	25 (73.5%)
- **NA**	9 (2.7%)	0 (0%)	9 (26.5%)

Supplementary Table S2: Description of auto-antibody testing among T1D, DIA2, DIA3, DIA4+ and MODY cohorts.

	T1D	DIA2	DIA3	DIA4+	MODY
**Antibodies Tested**					
- **Yes**	325 (96.4%)	17 (100%)	21 (80.8%)	22 (78.6%)	25 (73.5%)
- **No**	1 (0.3%)	0 (0%)	1 (3.8%)	0 (0%)	9 (26.5%)
- **NA**	11 (3.4%)	0 (0%)	4 (15.4%)	6 (21.4%)	0 (0%)
**AB (All)**					
- **Present**	322 (95.5%)	10 (58.8%)	14 (53.8%)	11 (39.3%)	0 (0%)
- **Absent**	2 (0.6%)	7 (41.2%)	7 (26.9%)	11 (39.3%)	25 (73.5%)
- **NA**	13 (3.9%)	0 (0%)	5 (19.2%)	6 (21.4%)	9 (26.5%)
**AAB-GAD65**					
- **Present**	246 (73.0%)	5 (29.4%)	10 (38.5%)	9 (32.1%)	0 (0%)
- **Absent**	82 (24.3%)	12 (70.6%)	16 (61.5%)	19 (67.9%)	25 (73.5%)
- **NA**	9 (2.7%)	0 (0%)	0 (0%)	0 (0%)	9 (26.5%)
**AAB-IA2**					
- **Present**	239 (70.9%)	8 (47.1%)	11 (42.3%)	6 (21.4%)	0 (0%)
- **Absent**	89 (26.4%)	9 (52.9%)	15 (57.7%)	22 (78.6%)	25 (73.5%)
- **NA**	9 (2.7%)	0 (0%)	0 (0%)	0 (0%)	9 (26.5%)
**AAB-Ins**					
- **Present**	87 (25.8%)	6 (35.3%)	5 (19.2%)	1 (3.6%)	0 (0%)
- **Absent**	241 (71.5%)	11 (64.7%)	21 (80.8%)	27 (96.4%)	25 (73.5%)
- **NA**	9 (2.7%)	0 (0%)	0 (0%)	0 (0%)	9 (26.5%)
**AAB-Znt8**					
- **Present**	0 (0%)	0 (0%)	0 (0%)	1 (3.6%)	0 (0%)
- **Absent**	328 (97.3%)	17 (100%)	26 (100%)	27 (96.4%)	25 (73.5%)
- **NA**	9 (2.7%)	0 (0%)	0 (0%)	0 (0%)	9 (26.5%)
